# Agonists and antagonists induce different palonosetron dissociation rates in 5-HT_3_A and 5-HT_3_AB receptors^☆^

**DOI:** 10.1016/j.neuropharm.2013.05.010

**Published:** 2013-10

**Authors:** Sarah C.R. Lummis, Andrew J. Thompson

**Affiliations:** Department of Biochemistry, University of Cambridge, Tennis Court Road, Cambridge CB2 1QW, UK

**Keywords:** Serotonin receptor, Allosteric binding site, Site-directed mutagenesis, Radioligand binding, FlexStation assays, 5-HT, 5-hydroxytryptamine (serotonin), HEK, human embryonic kidney, IC_50_, concentration of antagonist required for half-maximal inhibition, *K*_d_, affinity constant

## Abstract

Palonosetron is a potent 5-HT_3_ receptor antagonist with a unique structure and some unusual properties. Here we explore the properties of palonosetron at heterologously expressed 5-HT_3_A and 5-HT_3_AB receptors. We used receptors expressed in HEK293 cells, and functionally analysed them using a membrane potential sensitive dye in a Flexstation, which revealed IC_50_s of 0.24 nM and 0.18 nM for 5-HT_3_A and 5-HT_3_AB receptors respectively. Radioligand binding studies with [^3^H]palonosetron revealed similar K_d_s: 0.34 nM for 5-HT_3_A and 0.15 nM for 5-HT_3_AB receptors. Kinetic studies showed palonosetron association and dissociation rates were slightly faster in 5-HT_3_AB than 5-HT_3_A receptors, and for both subtypes dissociation rates were ligand-dependent, with antagonists causing more rapid dissociation than agonists. Similar ligand effects were not observed for [^3^H]granisetron dissociation studies. These data support previous studies which show palonosetron has actions distinct to other 5-HT_3_ receptor antagonists, and the slow rates observed for agonist induced dissociation (*t*_1/2_ > 10 h) could at least partly explain the long duration of palonosetron effects *in vivo*.

## Highlights

• Palonosetron IC_50_s and K_d_s for 5-HT_3_A and 5-HT_3_AB receptors in HEK293 cells are similar.
• Palonosetron association and dissociation rates are slower at 5-HT_3_A than 5-HT_3_AB receptors.
• Agonist-induced palonosetron dissociation rates are slower than those for antagonists in both 5-HT_3_A and 5-HT_3_AB receptors.
• Agonist- and antagonist-induced granisetron dissociation rates are similar in both 5-HT_3_A and 5-HT_3_AB receptors.
• Palonosetron and granisetron have distinct actions.

## 1 Introduction

**Fig. 1:**
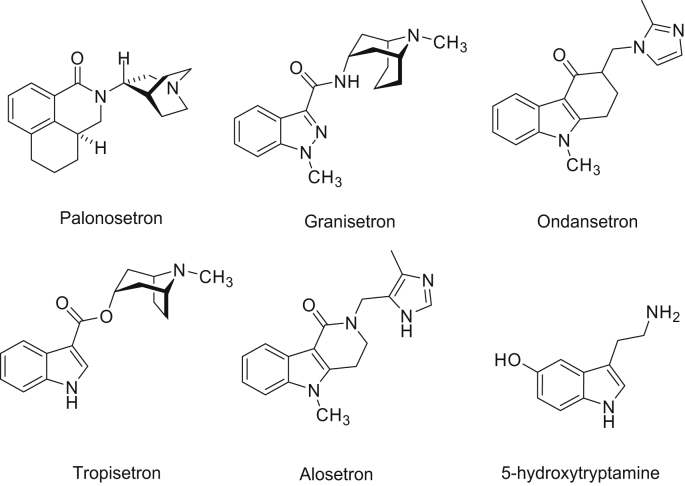
Chemical structures of 5-HT and clinically used 5-HT_3_ receptor competitive antagonists.

5-HT_3_ receptors are members of the Cys-loop family of ligand-gated ion channels, membrane proteins that are responsible for fast excitatory and inhibitory synaptic neurotransmission in central and peripheral nervous systems, and the targets for a number of important therapeutics. 5-HT_3_ receptor antagonists are routinely used in the management of post-operative, radiotherapy-induced and chemotherapy-induced nausea and vomiting and for treating irritable bowel syndrome in patients that do not respond adequately to conventional therapies. A range of other therapeutic applications has also been suggested (reviewed in (Thompson and Lummis, 2007; Walstab et al., 2010)). Antagonists are often referred to as the “setrons”, and include ondansetron, granisetron and palonosetron (Fig. 1). These drugs are potent (*K*_d_ = nM − pM), long lived *in vivo*, and most are highly selective for the 5-HT_3_ receptor. Furthermore, they are usually well tolerated and display only mild, transient side-effects, making them the preferred choice of drug in most instances (Aapro, 2004; Blower, 1995; Eglen et al., 1995; Hirata et al., 2007).

Palonosetron has a different structure from the other 5-HT_3_ antagonists (Fig. 1), and some distinctive properties. The first published accounts of these properties were in 1995, when radioligand binding experiments demonstrated that it bound to 5-HT_3_ receptors with high potency and selectivity, and *in vivo* data showed an anti-emetic efficacy greater than or equal to that of ondansetron or granisetron (Bonhaus et al., 1995; Eglen et al., 1995; Wong et al., 1995). At that time, however, it was not clear that there are multiple 5-HT_3_ receptor subunits, (A-E), in addition to alternative splice variants, thus providing the potential for a wide range of different 5-HT_3_ receptor subtypes. Heteromeric assemblies of 5-HT3A plus 5-HT3C, 5-HT3D or 5-HT3E subunits have not yet been extensively studied, but their biophysical properties appear similar to homomeric 5-HT_3_A receptors (see (Niesler, 2011) and (Walstab et al., 2010) for reviews). 5-HT_3_AB receptors, however, have been extensively investigated in heterologous systems, and have differing concentration–response curves (increased *EC*_50_ values and shallower Hill slopes), increased single channel conductance (5-HT_3_A = sub-pS; 5-HT_3_AB = 16–30 pS), an increased rate of desensitisation, reduced Ca^2+^ permeability and a non-linear current–voltage relationship (Davies et al., 1999; Kelley et al., 2003; Livesey et al., 2008). To determine if there are differences in the affinity and association and dissociation rates of palonosetron in 5-HT_3_A and 5-HT_3_AB receptors, we here explore the effects of palonosetron on 5-HT_3_ receptor function and binding in these receptor subtypes.

## 2 Materials and methods

### 2.1 Materials

All cell culture reagents were obtained from Gibco BRL (Paisley, U.K.), except foetal calf serum which was from Labtech International (Ringmer, U.K.). [^3^H]granisetron (84 Ci mmol^−1^) was from PerkinElmer (Boston, Massachusetts, USA). [^3^H]-palonosetron (37.2 Ci/mmol) was custom synthesised for Helsinn Healthcare (Lugano, Switzerland), and both this and the unlabelled form of palonosetron were kindly gifted by Helsinn Healthcare (Lugano, Switzerland). All other reagents were of the highest obtainable grade.

### 2.2 Cell culture and transfection

Human embryonic kidney (HEK) 293 cells were maintained on 90 mm tissue culture plates at 37 °C and 7% CO_2_ in a humidified atmosphere. They were cultured in DMEM:F12 (Dulbecco's Modified Eagle Medium/Nutrient Mix F12 (1:1)) with GlutaMAX™ I media containing 10% foetal calf serum and passaged when confluent. For radioligand binding studies cells in 90 mm dishes were transfected using PEI and incubated for 3–4 days before use. For functional studies cells were plated on 96 well plates, transfected using the Neon transfection system (Invitrogen) and incubated 1–2 days before assay. Mutagenesis reactions were performed using QuikChange (Agilent Technologies Inc., CA, USA) using human 5-HT_3A_ or 5-HT_3B_ receptor subunit cDNA (accession numbers: P46098 or O95264) in pcDNA3.1 (Invitrogen, Paisley, UK). Subunit numberings have been altered to the aligning residues in the mouse 5-HT_3_A receptor.

### 2.3 Radioligand binding

Methods were as previously described (Lummis et al., 1993), with minor modifications. Briefly, transfected HEK293 cells were washed twice with phosphate buffered saline (PBS) at room temperature and scraped into 1 ml of ice-cold HEPES buffer (10 mM, pH 7.4) containing the following proteinase inhibitors (PI): 1 mM EDTA, 50 μg ml^−1^ soybean trypsin inhibitor, 50 μg/ml bacitracin and 0.1 mM phenylmethylsulphonyl fluoride. Cells were homogenised, freeze-thawed, washed with HEPES buffer, and 50 μg of the crude cell membrane preparation incubated in 0.5 ml HEPES buffer containing [^3^H]granisetron or [^3^H]palonosetron at a range of concentrations for saturation binding, or at 0.3 nM and 0.1 nM respectively for competition binding and association/dissociation studies. Non-specific binding was determined using 10 μM quipazine. Equilibrium reactions were incubated for at least 1 h or 24 h for [^3^H]granisetron or [^3^H]palonosetron respectively at 4 °C. Dissociation was initiated with unlabelled ligands to give a final concentrations of 100 μM (5-HT), 10 μM (quipazine), 1 μM (MDL72222) or 100 nM (palonosetron). All samples were terminated by vacuum filtration using a Brandel cell harvester onto GF/B filters pre-soaked in 0.3% polyethyleneimine. Radioactivity was determined by scintillation counting using a Beckman LS6000SC (Fullerton, California, USA).

### 2.4 Fluorescent studies

**Fig. 2:**
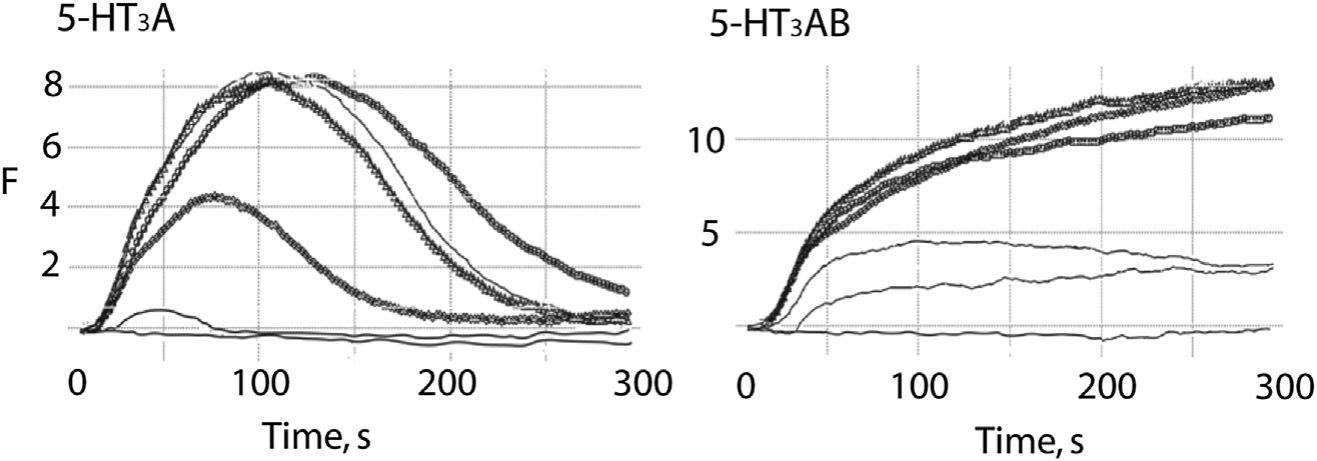
Typical Flexstation responses of HEK293 cells expressing 5-HT_3_A and 5-HT_3_AB receptors. 5-HT at various concentrations (0–30 μM) was added at 20 s. Note the shapes of the responses, which are different in homomeric and heteromeric receptors. F = arbitrary fluorescent units.

These were performed as previously described (Price and Lummis, 2005). Briefly, cells were gently rinsed twice with buffer (10 mM HEPES, 115 mM NaCl, 1 mM KCl, 1 mM CaCl_2_, 1 mM MgCl_2_, 10 mM glucose, pH 7.4) and 100 μl fluorescent membrane-potential sensitive dye (Molecular Devices) added. Cells were then incubated at room temperature for 45 min before assay. For inhibition studies, palonesetron was added either with the dye, ensuring a 45 min pre-incubation, or simultaneously with 5-HT (co-application). Fluorescence was measured in a FLEXstation™ (Molecular Devices Ltd., Wokingham, UK) every 2 s for 200 s using the acquisition software SOFTmax^®^ PRO v4.3. Control (buffer alone) or 5-HT (0.001 μM–30 μM) was added to each well at 20 s. Typical responses are shown in Fig. 2.

### 2.5 Data analysis

Data were analysed by iterative curve fitting using Prism software (GraphPad, San Diego, California, USA). Determination of K_i_ values was performed using the Cheng–Prusoff equation. Values are presented as mean ± SEM, *n* = 3–6.

## 3 Results

### 3.1 Functional studies

**Fig. 3:**
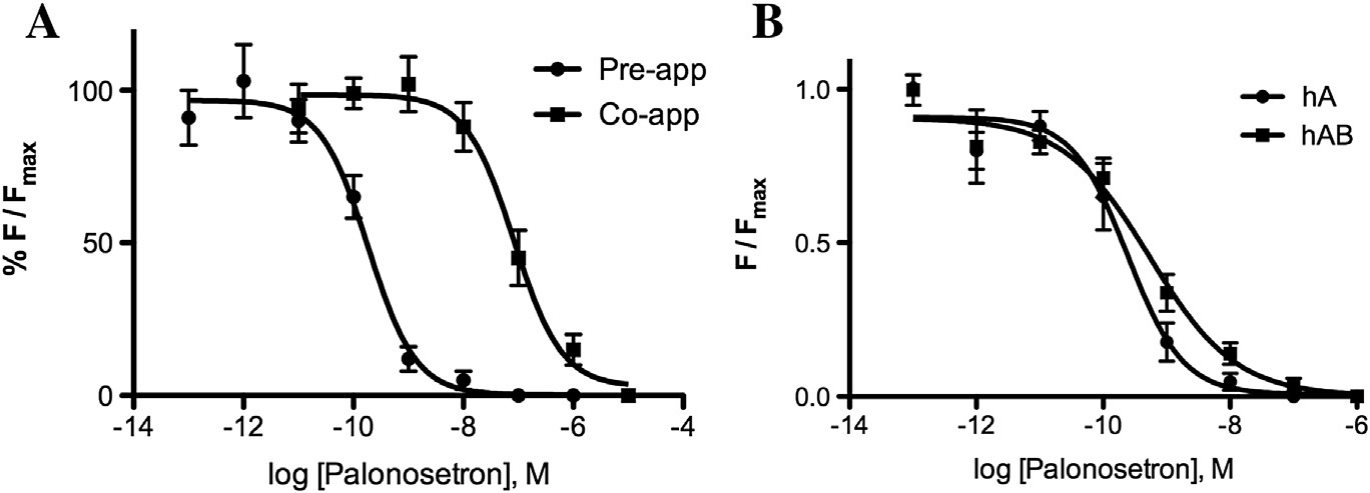
Inhibition of 5-HT-induced responses in HEK293 cells. A: Palonosetron is more potent when pre-applied () than when co-applied () with 5-HT; example in cells expressing 5-HT_3_A receptors. B. Palonosetron has similar IC_50_s at 5-HT_3_A and 5-HT_3_AB receptors. Parameters derived from these data are given in Section 3.1.

Examination of palonosetron inhibition of 5-HT_3_ receptors expressed in oocytes revealed very slow recovery after washout, with <10% of the original response being recovered after a 10 min wash (data not shown). We therefore determined the inhibitory effects of palonosetron on 5-HT-induced responses using 5-HT_3_ receptors expressed in HEK293 cells loaded with membrane sensitive fluorescent dye where washout is not required. Preliminary experiments revealed palonosetron required at least 5 min incubation before application of 5-HT to reveal its full inhibition, and thereafter it was preincubated for 45 min. Data revealed different apparent potencies of palonosetron at 5-HT_3_A receptors, depending upon whether it was preincubated (pIC_50_ = 9.73 ± 0 0.13; IC_50_ = 0.18 nM, *n* = 4) or co-applied with 5-HT (pIC_50_ = 7.08 ± 0.14; IC_50_ = 83 nM, *n* = 4), suggesting that palonosetron has a relatively slow on rate, and in particular that its on rate is slower than that of 5-HT (Fig. 3A).

With a 45 min preincubation, palonosetron inhibition of 5-HT-induced responses (Fig. 3B) revealed sub-nanomolar IC_50_s at both 5-HT_3_A and 5-HT_3_AB receptors: pIC_50_ = 9.61 ± 0.13; (IC_50_ = 0.24 nM), and 9.73 ± 0.27; (IC_50_ = 0.18 nM) respectively (*n* = 4).

**Fig. 4:**
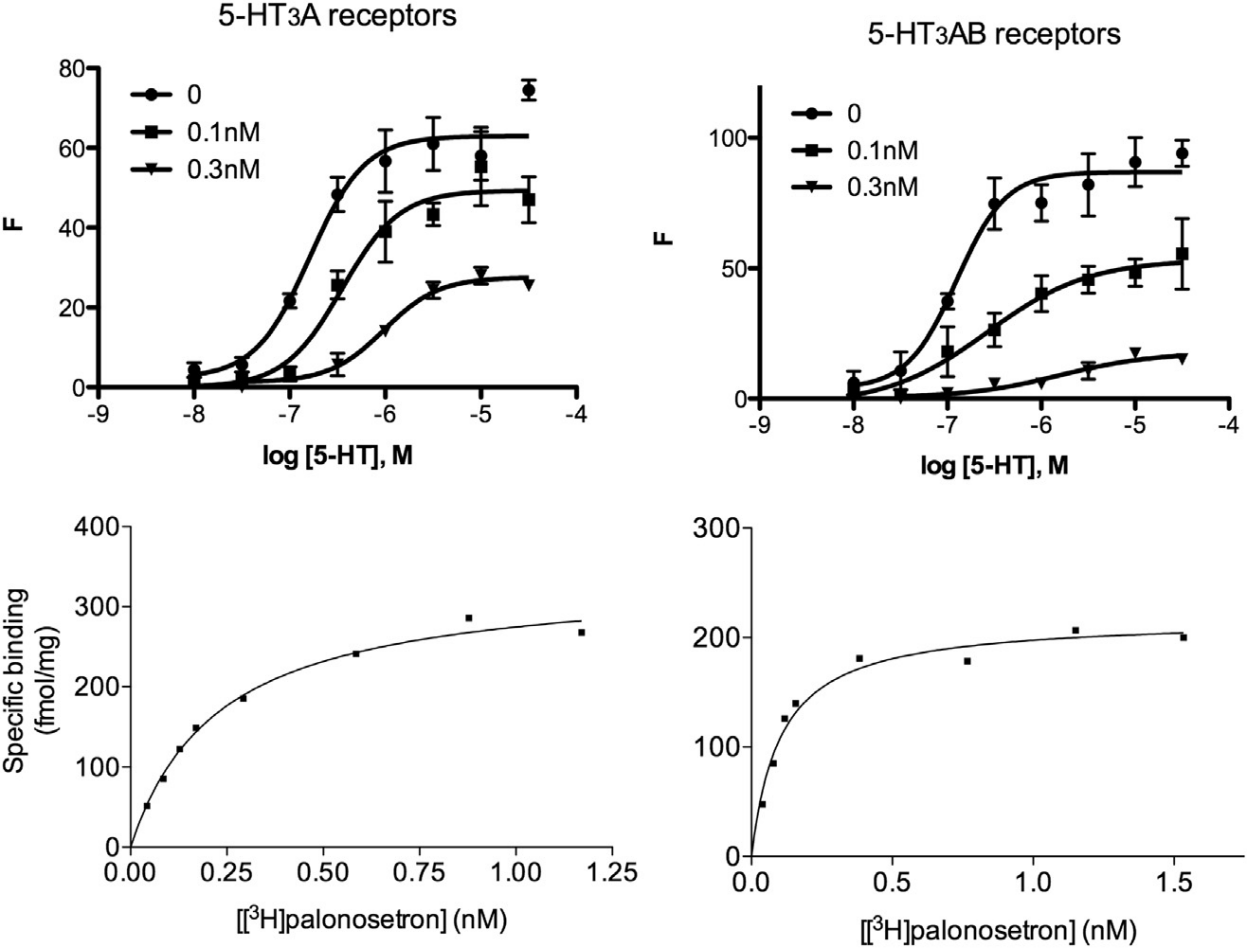
Functional and radioligand binding data suggest similar effects of palonosetron at 5-HT_3_A and 5-HT_3_AB receptors. Top: Typical Flexstation data from 5-HT_3_A and 5-HT_3_AB receptor-expressing cells; EC_50_ values increase and *F*_max_ values decrease as [palonosetron] increases. In this typical example EC_50_s are 0.16 μM, 0.35 μM and 0.97 μM with relative *F*_max_ values of 100%, 70% and 40% for 0, 0.1 nM and 0.3 nM palonosetron in 5-HT_3_A receptors, and 0.12 μM (100%), 0.27 μM (62%) and 1.6 μM (28%) in 5-HT_3_AB receptors. Lower panel: typical radioligand binding curves for 5-HT_3_A and 5-HT_3_AB receptors.

Examination of 5-HT concentration response curves in the presence of 0, 0.1 nM or 0.3 nM palonosetron revealed increased EC_50_s and decreased maximal responses with increasing palonosetron concentrations (Fig. 4).

### 3.2 Radioliogand binding

Palonosetron displacement of the 5-HT_3_-specific ligand [^3^H]granisetron revealed similar potencies at 5-HT_3_A and 5-HT_3_AB receptors. pIC_50_s = 9.22 ± 0.05; (IC_50_ = 0.60 nM; *K*_i_ = 0.3 nM), and 9.14 ± 0 0.18 (IC_50_ = 0.71 nM; *K*_i_ = 0.35 nM) respectively. Saturation experiments with a range of [^3^H]palonosetron concentrations revealed high affinity binding with *K*_d_ values of 0.34 ± 0.04 and 0.15 ± 0.04 nM at 5-HT_3_A and 5-HT_3_AB receptors respectively. Typical data is shown in Fig. 4.

### 3.3 Association and dissociation rates

Association of [^3^H]palonosetron was complete in ∼30 min at both 5-HT_3_A and 5-HT_3_AB receptors, with *t*_1/2_ values of 4.1 (*k* = 0.16 ± 0.03) and 2.0 min (*k* = 0.35 ± 0.06) respectively (significantly different, *t*-test, *p* < 0.05). This is slower than previously reported where association was complete in under 10 min (Wong et al., 1995).

**Fig. 5:**
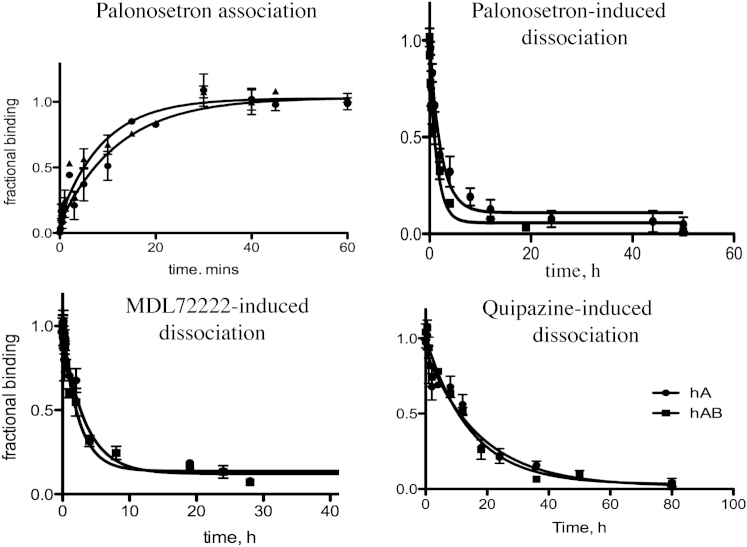
Association and dissociation curves for [^3^H]palonosetron using HEK293 cells expressing 5-HT_3_A and 5-HT_3_AB receptors. Association was rapid for both 5-HT_3_A and 5-HT_3_AB receptors, with maximal levels being reached within 30 min. Dissociation rates were slower, although were faster with antagonists (MDL72222, palonosetron) than agonists (quipazine). Parameters derived from these data are given in Section 3.3.

**Fig. 6:**
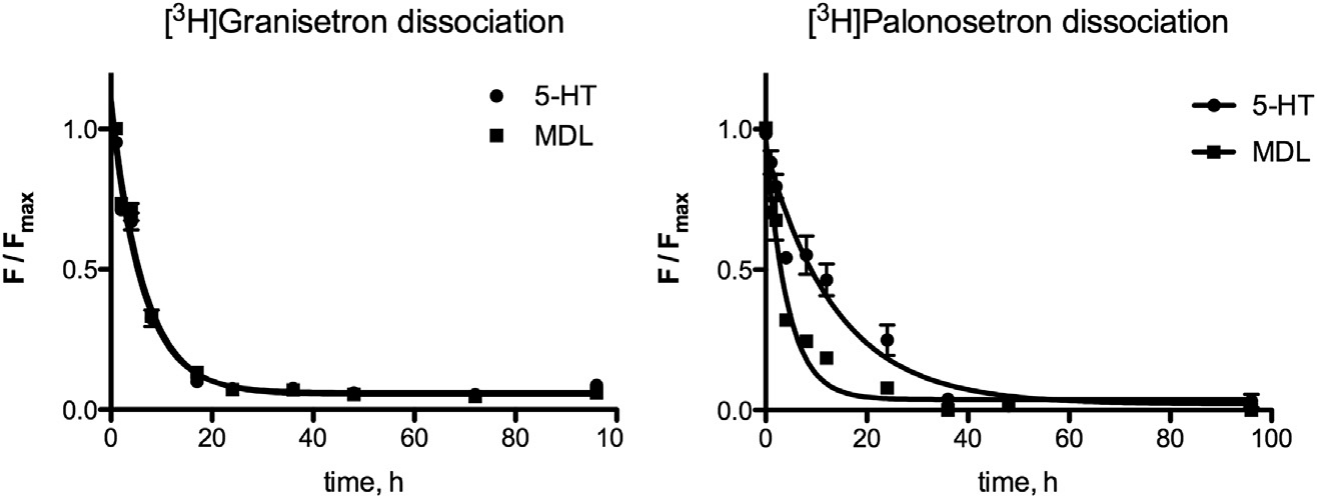
[^3^H]granisetron and [^3^H]palonosetron dissociation curves in 5-HT_3_A receptors. Dissociation curves for [^3^H]granisetron using HEK293 cells expressing 5-HT_3_A receptors using 5-HT (agonist) or MDL72222 (antagonist) are superimposable. However data from [^3^H]palonosetron dissociation experiments reveal a clear distinction between agonist and antagonist displacement rates. Data from these curves are shown in Table 1.

Our dissociation rates were also slower than previously reported, and were also strongly dependent on the ligand used for dissociation. An excess of unlabelled palonosetron gave *t*_1/2_ values for dissociation of 1.5 h and 1.0 h for 5-HT_3_A and 5-HT_3_AB receptors respectively, with values of 2.3 h and 1.7 h when an excess of MDL72222 was used. However when the agonists 5-HT or quipazine were used, *t*_1/2_ values were >10 h. Data are shown in Table 1 with example curves in Fig. 5. These reveal that in the presence of agonists specific binding does not approach background levels until 2–5 days after dissociation is initiated. Similar experiments using [^3^H]granisetron revealed no differences in rates using agonists or antagonists (Table 1 and Fig. 6).

## 4 Discussion

Palonosetron is a potent 5-HT_3_ receptor antagonist which is proving to be superior to other 5-HT_3_ receptor antagonists for the treatment of acute and delayed post-operative, radiotherapy-induced and chemotherapy-induced nausea and vomiting. The unusual properties of palonosetron, which have been proposed to explain its superiority, include allosteric interactions, positive co-operativity and palonosetron-triggered 5-HT_3_ receptor internalization (Rojas et al., 2008, 2010). Internalization was not explored in our association and dissociation rate experiments, as this process would not occur at the temperatures at which these experiments were performed, but the slow palonosetron dissociation rate shown here could provide an additional or alternative explanation to internalization: our data show palonosetron has the ability to inhibit 5-HT_3_ receptors for considerably longer than the more established 5-HT_3_ receptor antagonists such as ondansetron and granisetron, which could result in a prolonged anti-emetic behaviour.

Palonosetron is a potent inhibitor of 5-HT-induced increases in fluorescence of a membrane potential sensitive dye. Inhibition of 5-HT_3_ receptor function at low concentrations was expected as palonosetron has an affinity for 5-HT_3_ receptors that is greater than other commonly used 5-HT_3_ receptor antagonists (e.g. NG108-15 cells *K*_d_ = 0.05 nM (Wong et al., 1995), human hippocampus *K*_d_ = 0.15 nM (Wong et al., 1995), transfected HEK293 cells *K*_d_ = 0.2 nM (Rojas et al., 2008)). The slow dissociation rates we observed (*t*_1/2_ = 1–22 h) differ from previous studies (*t*_½_ = 8–10 min) (Wong et al., 1995), but are consistent with the slow recovery from palonosetron-induced 5-HT_3_ receptor inhibition we observed in our preliminary experiments in oocytes, which precluded us from using them in this study. Somewhat similar data were observed by Rojas et al. (2008), who showed that 53 ± 11% of [^3^H]palonosetron remained associated with 5-HT_3_ receptor-expressing HEK293 cells after a 2.5 h wash. This is akin to our data: e.g. dissociation of [^3^H]palonosetron from 5-HT_3_A receptors in the presence of MDL72222 resulted in 67 ± 7% (*n* = 4) of [^3^H]palonosetron remaining after 2 h. Subsequent experiments by Rojas et al. (2010) suggested a difference between cells and cell-free membranes, with 65% [^3^H]palonosetron remaining in cells after a 60 min wash, but only 2% remaining in a cell-free membrane preparation (Rojas et al., 2010). Our preparation is similar to their cell free preparation, (i.e. it does not contain whole cells) although it is not washed as extensively, and our cells were not treated with trypsin; thus it may be that the different procedures can significantly effect binding characteristics.

We did not observe any major difference between the effects of palonosetron at homomeric (5-HT_3_A) and heteromeric (5-HT_3_AB) receptors. 5-HT_3_A receptors may predominate in the CNS, while 5-HT_3_AB receptors may be more abundant in the PNS. Nevertheless, both 5-HT3A and 5-HT3B subunits, and indeed the other three subunits in this family (5-HT3C–5-HT3E), are widely distributed in many body regions (Holbrook et al., 2009; Niesler, 2011). Our data indicate similar palonosetron *K*_d_s at 5-HT_3_A and 5-HT_3_AB receptors, consistent with previous work showing that competitive antagonists (with one exception, see below) do not show major differences in potency between these two receptor subtypes. This is because the binding site for these compounds is located between two 5-HT_3_A subunits, which assemble as part of the 5-HT_3_AB pentamer (Lochner and Lummis, 2010; Thompson et al., 2011). The only competitive antagonist that has been identified with distinct affinities at 5-HT_3_A and 5-HT_3_AB receptors is VUF10166, and the different in affinities is due to an allosteric binding site at an A + B- interface (Thompson et al., 2012). An allosteric mechanism has also been previously suggested for palonosetron (Moura Barbosa et al., 2010) using computational data, and allosteric binding characteristics have been reported (concave Scatchard plots and Hill slopes of 1.5 (Rojas et al., 2008). Our data showing increased EC_50_s and decreased maximal responses with increasing concentrations of palonosetron are consistent with a non-competitive mode of action of this compound, i.e. action at an allosteric site. However they are also consistent with an irreversible competitive antagonist, and, given the slow off rates of [^3^H]palonosetron, we consider this is the correct interpretation, and that these data reflect the fact that palonosetron does not significantly dissociate from the receptor during these experiments.

We did observe a difference in [^3^H]palonosetron dissociation rates depending on the unlabelled ligand used for displacement, with antagonists resulting in more rapid dissociation compared to agonists; similar findings have been previously observed for native 5-HT_3_ receptors in NG108-15 cells, and 5-HT_3_A receptors in HEK293 cells (Bonhaus et al., 1995). These authors suggest that binding of agonists to unoccupied binding sites can increase the receptors affinity for prebound ligands and thereby slow their dissociation. We propose there is a similar mechanism in our experiments. At the start of the dissociation experiments up to five potential binding sites could be occupied by [^3^H]palonosetron in 5-HT_3_A receptors, with less (1–3) in 5-HT_3_AB receptors. Subsequent occupancy of a binding site by unlabelled palonosetron or another competitive antagonist such as MDL72222 would not alter the state of the receptor, while agonist binding likely causes entry into a high affinity desensitized state. Palonosetron, in one or more of the remaining binding sites, would then only dissociate slowly.

Our data also show that both association and dissociation rates are slightly faster for 5-HT_3_AB receptors, which may be due to subtle difference in structure of this receptor; it is known, for example, that these receptors are more prone to spontaneous opening (Hu and Peoples, 2008). Our data reveal that agonists have a similar effect in homomeric and heteromeric receptors, providing evidence that there is more than one orthosteric (A + A-) binding site in heteromeric receptors.

In conclusion we have shown that palonosetron binds with similar affinities at 5-HT_3_A and 5-HT_3_AB receptors. We observed that in both receptor subtypes there is slow dissociation of [^3^H]palonosetron, and its rate is ligand-dependent. This slow dissociation, which is particularly pronounced in the presence of agonists, provides a possible additional or alternative explanation for the long lasting therapeutic effects of palonosetron. Our conclusions are strongly supported by a study published during revision of this manuscript (Hothersall et al., 2013). These authors used [^3^H]granisetron binding and ELISA to monitor COS-7 cells transfected with 5-HT_3_ receptors. Their data, obtained predominantly from live cells incubated at a variety of temperatures, indicate that palonosetron acts as a pseudo-irreversible antagonist causing prolonged inhibition due to slow dissociation, with no contribution from endocytosis.

## Figures and Tables

**Table 1:** [^3^H] palonosetron and [^3^H] granisetron binding dissociation rates with different displacing ligands using 5-HT_3_ receptors expressed in HEK293 cells.* = significantly different to 5-HT_3_A receptors, *t*-test, *p* < 0.05.

	5-HT_3_A receptors		5-HT_3_AB receptors	
	k_−1_ (mean ± S.E.M)	t_1/2_ (h)	k_−1_ (mean ± S.E.M)	t_1/2_ (h)
**[**^**3**^**H] palonosetron**				
5-HT	0.032 ± 0.005	21.9	0.057 ± 0.008*	12.1
Quipazine	0.056 ± 0.003	12.5	0.068 ± 0.003*	10.1
MDL72222	0.29 ± 0.03	2.3	0.41 ± 0.04*	1.7
Palonosetron	0.44 ± 0.05	1.5	0.68 ± 0.08*	1.0
**[**^**3**^**H] granisetron**				
5-HT	0.14 ± 0.020	4.6	0.17 ± 0.023	4.0
MDL72222	0.15 ± 0.023	4.8	0.16 ± 0.021	4.2
